# Nonsense-Mediated mRNA Decay Impacts MSI-Driven Carcinogenesis and Anti-Tumor Immunity in Colorectal Cancers

**DOI:** 10.1371/journal.pone.0002583

**Published:** 2008-07-09

**Authors:** Jamila El-Bchiri, Agathe Guilloux, Peggy Dartigues, Etienne Loire, Dominique Mercier, Olivier Buhard, Iradj Sobhani, Pierre de la Grange, Didier Auboeuf, Françoise Praz, Jean-François Fléjou, Alex Duval

**Affiliations:** 1 INSERM, UMR S893, Team 13 “Microsatellite Instability and Cancers”, Paris, France; 2 UPMC Univ Paris 06, UMR S893, Paris, France; 3 UPMC Univ Paris 06, Atelier de Bioinformatique, Paris, France; 4 UPMC Univ Paris 06, UMR 7592, Institut Jacques Monod, Paris, France; 5 Département de Gastro-Entérologie, CHU Henri Mondor, Créteil, France; 6 INSERM, U685, Hôpital Saint-Louis, Paris, France; University of Cape Town, South Africa

## Abstract

Nonsense-mediated mRNA Decay (NMD) degrades mutant mRNAs containing premature termination codon (PTC-mRNAs). Here we evaluate the consequence of NMD activity in colorectal cancers (CRCs) showing microsatellite instability (MSI) whose progression is associated with the accumulation of PTC-mRNAs encoding immunogenic proteins due to frameshift mutations in coding repeat sequences. Inhibition of UPF1, one of the major NMD factors, was achieved by siRNA in the HCT116 MSI CRC cell line and the resulting changes in gene expression were studied using expression microarrays. The impact of NMD activity was also investigated in primary MSI CRCs by quantifying the expression of several mRNAs relative to their mutational status and to endogenous UPF1 and UPF2 expression. Host immunity developed against MSI cancer cells was appreciated by quantifying the number of CD3ε-positive tumor-infiltrating lymphocytes (TILs). UPF1 silencing led to the up-regulation of 1251 genes in HCT116, among which a proportion of them (i.e. 38%) significantly higher than expected by chance contained a coding microsatellite (*P*<2×10^−16^). In MSI primary CRCs, UPF1 was significantly over-expressed compared to normal adjacent mucosa (*P*<0.002). Our data provided evidence for differential decay of PTC-mRNAs compared to wild-type that was positively correlated to UPF1 endogenous expression level (*P* = 0.02). A negative effect of UPF1 and UPF2 expression on the host's anti-tumor response was observed (*P*<0.01). Overall, our results show that NMD deeply influences MSI-driven tumorigenesis at the molecular level and indicate a functional negative impact of this system on anti-tumor immunity whose intensity has been recurrently shown to be an independent factor of favorable outcome in CRCs.

## Introduction

Nonsense-mediated mRNA decay (NMD) is an evolutionarily conserved mRNA surveillance mechanism that recognizes and eliminates aberrant mRNAs harboring premature termination codons (PTC), thereby preventing the accumulation of potentially deleterious truncated proteins in eukaryotic cells [1]. Following pre-mRNA splicing, NMD targets are recognized via a multiprotein exon-junction complex (EJC) that is deposited 24 nucleotides upstream of each exon-exon junction. As a general rule, it has been established that aberrant mRNAs containing PTC located either less than 50–55 nucleotides upstream of the last exon-exon junction or in the last exon are not degraded by NMD (NMD-irrelevant) [2]. The core of NMD effectors comprises the evolutionary-conserved UPF proteins, UPF1/RENT1, UPF2/RENT2, and two paralogs of UPF3, UPF3 (also called UPF3a) and UPF3X (also called UPF3b). UPF1 is an RNA helicase whose activity is regulated by cycles of phosphorylation/dephosphorylation. Phosphorylation of UPF1 requires UPF2 and UPF3 and is catalyzed by SMG1, a protein kinase related to the phosphoinositide-3-kinase family. UPF1 phosphorylation by SMG1 has been shown to be the rate-limiting step in NMD [3], [4]. It is noteworthy that SMG1 activity is not only devoted to NMD but also plays a role in DNA damage signaling and repair, notably by phosphorylating p53 [5]. Dephosphorylation of UPF1 is mediated by SMG5, SMG6 and SMG7, three proteins that act as adaptors between phosphorylated UPF1 and protein phosphatase 2A [3]. UPF1 function is crucial in NMD, while UPF3 and UPF3X are partially redundant [6] and UPF2 is dispensable in some cases suggesting the existence of a UPF2-independent NMD pathway [7]. UPF1 acts not only in NMD-mediated degradation of aberrant transcripts but also in the physiological decay of various mRNAs, regulating the expression of 3–10% of the transcriptome [8], [9]. Silencing of UPF1 and to a lesser extent UPF2 has been reported to modulate the expression level of a number of physiological substrates of NMD, including transcripts with upstream open reading frames in the 5′-untranslated region, transcripts containing an intron within their 3′-untranslated region as well as transcripts derived from transposon or endogenous retrovirus [8], [10]. NMD has also been proposed to play a role in the regulation of alternative splicing events, since sequence-based analyses predict that about 35% of mammalian alternative splicing events produce PTC-containing spliced variants. Nevertheless, it has recently been reported that most PTC-containing splice variants are produced at low levels in human cells, independently of the action of NMD, suggesting that the majority of such PTC-introducing events are not under positive selection pressure and therefore are not expected to contribute important functional roles [11].

To date, the consequences of NMD activity have been mostly investigated in monogenic hereditary diseases, including hereditary tumors [12]. NMD has been reported to protect heterozygous carriers from the deleterious effects of some aberrant PTC-mRNAs encoding truncated proteins with dominant-negative activity [12]. Conversely, NMD inability in degrading NMD-irrelevant transcripts has been proposed to favor the phenotypic expression of dominant hereditary diseases [12]. Because cancer cells are genetically unstable and accumulate numerous somatic frameshift mutations in genes with an expected role in cell transformation, the NMD system has been proposed to play a role in tumor development [12]. However, the assessment of its overall impact on this process has been poorly described and remains hard to define since it depends on the nature and the number of mutants accumulated in cancer cells during tumor progression. To date, NMD inhibition has been successfully used as an *in vitro* strategy to discover new cancer-related genes harboring truncating mutations, suggesting that it may indeed have a role in favoring the selection of some frequent mutational events in various tumor types [13]. It is noteworthy that NMD activity has been shown to be highly variable, leading to incomplete and differential decay of putatively NMD-relevant mRNAs containing a PTC (referred to as NMD-escape) [14], [15], [16].

MSI tumors harboring mismatch repair (MMR) deficiency are frequent in humans. They represent about 15% of sporadic colorectal, gastric and endometrial tumors and include neoplasms arising in the Hereditary Non Polyposis Colorectal Cancer (HNPCC) syndrome. Dozens of mutations affecting genes containing coding repeat sequences have been reported in these tumors, defining the so-called mutator pathway whose role is suspected to be crucial in MSI-driven tumorigenesis [17]. To date, a large number of publications have reported frameshift mutations in genes involved in various biological pathways such as cell cycle regulation (e.g. *TGFBR2*, *IGF2R*, *TCF4*, *AXIN2*, *PTEN*, *RIZ*), apoptosis (e.g. *BAX*, *CASP5*, *BCL10*, *APAF1*, *FAS*), DNA damage repair (e.g. *ATR*, *DNA-PKcs*, *RAD50*, *MSH3*, *MSH6*, *MBD4*, *MLH3*, *BLM*, *CHK1*) and others [17]. We recently reported that the decay of frameshift mutation-derived mRNAs following *in vitro* silencing of UPF1 and/or UPF2 in a panel of MSI CRC cell lines was differential and incomplete [14]. If not fully degraded, frameshift mutant mRNAs encode proteins containing aberrant C-terminal tails, among which some have been shown to display immunogenic properties [18], [19]. Several studies have emphasized the fact that, in keeping with this process, MSI tumors were markedly infiltrated by cytotoxic intra-epithelial tumor-infiltrating T lymphocytes (TILs) and that such a cellular immune response was predictive of a relatively favorable outcome independently of the initial tumor stage and other clinical factors [20]. Therefore, NMD activity may interfere with anti-tumor immunity by limiting the expression of some of these aberrant proteins. Using MSI colorectal cancer as a model, we aimed here at further investigating the role of NMD in oncogenesis.

## Results

### Transcriptome changes secondary to UPF1 silencing in HCT116 CRC cells and their relationship to microsatellite instability

Using Affymetrix GeneChip® Human Exon 1.0 ST gene expression arrays, the expression of 1363 genes was found to be significantly deregulated upon UPF1 silencing in the HCT116 (MSI) CRC cell line, with a fold change ≥1.5 compared to untreated cells (Table S1). With 111 others, UPF1 was, as expected, one of the genes to be significantly down-regulated. The level of inhibition was significant (>75%) and agreed well with our data obtained by real-time quantitative RT-PCR (data not shown). Overall, 1251 genes were up-regulated upon UPF1 silencing (1251/1363, i.e. 92% of all deregulated genes under these conditions), amongst which 472 (38%) contained a mononucleotide repeat sequence of at least 7 base pairs in the coding region (Table S2). Of interest, the total number of genes in the human genome with a coding repeat tract ≥7N is lower (4470/22218 ; 20%. Data not shown), making significantly higher than expected by chance the overall number of such target genes up-regulated in HCT116 upon UPF1 silencing (*P*<2×10^−16^; Chi2 test).

**Figure 1:**
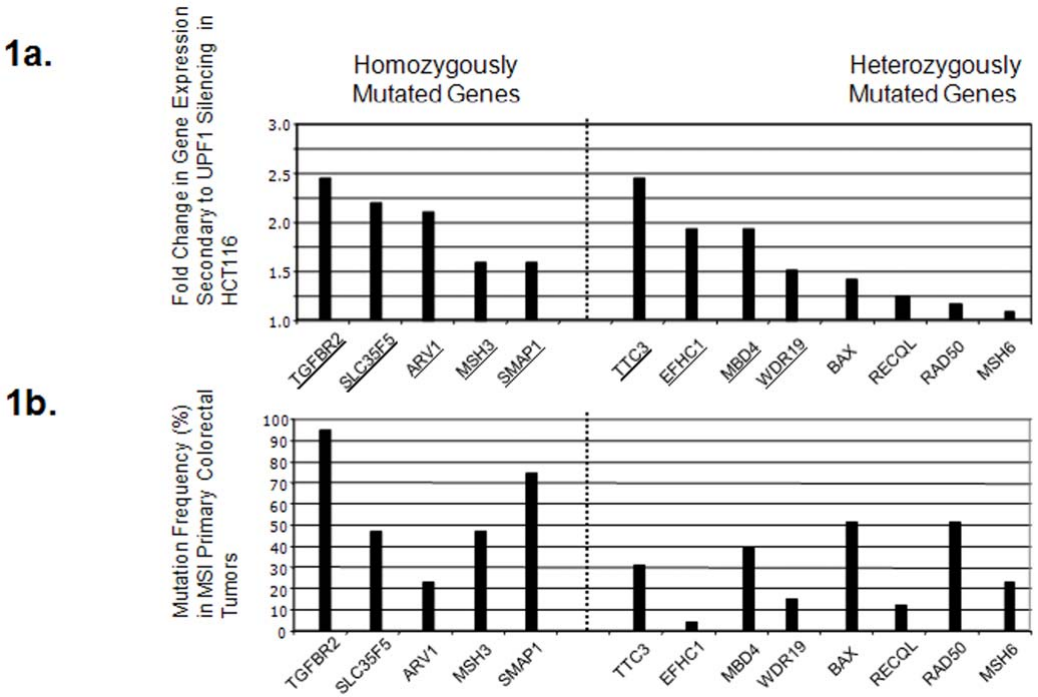
a. Impact of UPF1 on the decay of PTC-mRNAs in HCT116 cells. *TGFBR2*, *SLC35F5*, *ARV1*, *MSH3*, *SMAP1*, *TTC3*, *EFHC1*, *MBD4*, *WDR19*, *BAX*, *RECQL*, *RAD50* and *MSH6* harbor homozygous or heterozygous frameshift mutations in the HCT116 MSI CRC cell line. Using expression array, fold changes in the expression of the corresponding mRNAs relative to UPF1 silencing were determined (fold change = E_mRNA_ in cells treated with a UPF1 siRNA / E_mRNA_ in cells treated with control siRNA). Genes that are underlined are those whose re-expression was significant in these conditions (1.5 fold-change up with a p-value ≤0.005). Evidence for variable sensitivity to UPF1-mediated decay of such PTC-mRNAs could therefore be obtained, although allele specific expression assays were not used to differentiate between mutated and wild-type mRNAs in the case of heterozygous frameshift alterations. b. Coding frameshift mutations in target genes relative to NMD-status in MSI primary CRCs. Frequencies of frameshift mutations of the same series of 13 target genes for instability in 44 MSI primary CRCs are represented. Target genes whose frameshift mutations are frequently selected for during MSI CRC progression harbor different sensitivities to UPF1-mediated decay.

Amongst the aforementioned 472 genes in HCT116 cells, 22 genes containing a coding mononucleotide repeat of 7 to 10 nucleotides were chosen because of their putative role in colorectal carcinogenesis and screened for insertion/deletion mutations in these cells (Table 1). All these genes but 3 (*MBD4*, *MSH3*, *TGFBR2*) had never been reported to be mutated in MSI CRCs (Table 1). Using this approach, we detected or confirmed homozygous mutations in the *SLC35F5*, *TGFBR2*, *ARV1*, *MSH3*, *SMAP1* genes and heterozygous mutations in the *MBD4*, *EFHC1*, *TTC3*, and *WDR19* genes (Figure 1a). All the corresponding mutant mRNAs harbored a PTC located in coding regions that may be prone to NMD. In addition, we observed that 4 other target genes with previously described heterozygous frameshift mutations in HCT116 (*BAX*, *RECQL*, *RAD50* and *MSH6*) were not significantly re-expressed following UPF1 silencing (Figure 1a). The re-expression rates for PTC-mRNAs induced by UPF1 silencing in HCT116 are shown in Figure 1a and highlight the fact that, as described [14], UPF1-mediated mRNA decay is highly variable within a series of endogenously mutated PTC-mRNAs although allele specific expression assays were not used to differentiate between mutated and wild-type mRNAs in the case of heterozygous frameshift alterations.

**Table 1:** List of 22 Target genes up-regulated following UPF1 silencing in HCT116 and their mutational status.

Gene Symbol	Ensembl Human Gene	Fold Change	Repeat	Repeat Position in cDNA	Type of Mutation in HCT116
**ARV1**	**ENSG00000148926**	**2.08**	**9A**	**539**	**hmz**
CDC23	ENSG00000094880	1.99	7A	411	wt
CSPG2	ENSG00000038427	2.17	7A	7874	wt
**EFHC1**	**ENSG00000096093**	**1.94**	**7A**	**1316**	**htz**
MBD4*	ENSG00000129071	1.89	10A	930	htz
MSH3*	ENSG00000113318	1.6	8A	1394	hmz
NR3C1	ENSG00000113580	1.87	7A	1974	wt
ORC6L	ENSG00000091651	2.24	7A	561	wt
PIGB	ENSG00000069943	1.85	8T	1086	wt
PSD3	ENSG00000156011	1.6	8A, 8A	737, 2333	wt
PSEN1	ENSG00000080815	2.02	7T	774	wt
REV3L	ENSG00000009413	1.91	8A	1481	wt
RIF1	ENSG00000080345	1.75	7T, 8A	1538, 4637	wt
SHPRH	ENSG00000146414	1.69	8A	887	wt
**SLC35F5**	**ENSG00000115084**	**2.2**	**10T**	**1157**	**hmz**
**SMAP1**	**ENSG00000112305**	**1.6**	**10A**	**550**	**hmz**
TBC1D23	ENSG00000036054	1.66	9A	1901	wt
TFE3	ENSG00000068323	2.39	8G	1676	wt
TGFBR2*	ENSG00000163513	2.45	10A	831	hmz
TMEM161B	ENSG00000164180	1.57	8T	651	wt
**TTC3**	**ENSG00000182670**	**2.42**	**8A, 7A**	**1867, 2432**	**htz**
**WDR19**	**ENSG00000157796**	**1.54**	**8T**	**788**	**htz**

The threshold for re-expression was considered significant when the fold change was >1.5 (see the Materials and Methods section). Newly described target genes for instability in MSI CRCs are indicated in bold characters. Hmz: homozygously mutated at the coding repeat tract in HCT116. Htz: heterozygously mutated at the coding repeat tract in HCT116. wt: not mutated at the coding repeat tract in HCT116. Target genes that have been already described to be mutated in MSI CRCs are indicated with an ^*^.

### Frameshift mutations in MSI primary CRCs according to their NMD status

All the target genes mutated in HCT116 for which the impact of NMD had previously been determined were screened for frameshift mutations in coding microsatellite sequences in a series of primary MSI CRCs (n = 44). This includes genes whose mutated mRNAs are more or less sensitive to NMD (*TGFBR2*, *SLC35F5*, *ARV1*, *MSH3*, *TTC3*, *EFHC1*, *MBD4*, *SMAP1*, *WDR19*, *BAX*, *RECQL*, *RAD50*, *MSH6*) (Figure 1a). The mutation frequencies of these 13 genes representing possible targets for MSI-driven instability were highly variable in these tumors (Figure 1b). Based on their high mutation frequency, *SLC35F5*, *ARV1*, *TTC3*, and *SMAP1* represent new target genes in which frameshift mutations have not previously been reported in MSI CRC. They were mutated in 48% (21/44), 23% (10/44), 32% (14/44) and 73% (32/44) of tumors, respectively (Figure 1b).

### Over-expression of UPF1 mRNA in MSI primary CRCs

**Figure 2:**
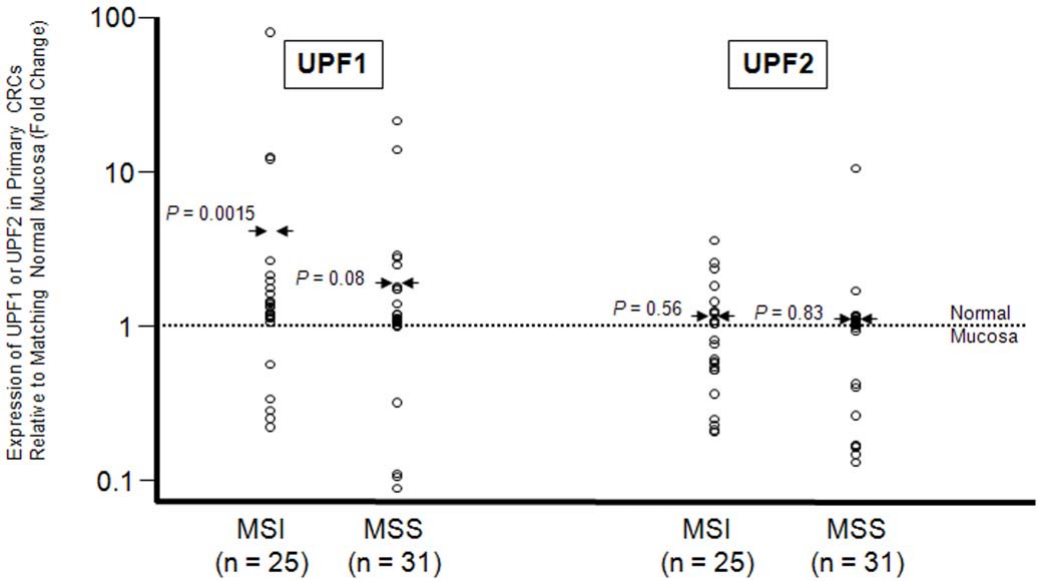
Expression of UPF1 and UPF2 in MSI and non-MSI (MSS) primary CRCs relative to matched normal colonic mucosa. In 25 MSI and 31 MSS CRCs we compared the expression of UPF1 and UPF2 between the tumors and matched normal colonic mucosa by real-time quantitative RT-PCR. In all but 5 cases, over-expression of UPF1 was observed in MSI CRC tumors. Considering all samples, the over-expression of UPF1 in MSI tumors was statistically significant (*P* = 1.5×10^−3^; paired t-test). In MSS CRCs, a trend for over-expression of this factor was observed under the same conditions (*P* = 0.08; paired t-test). In patients with MSI or MSS CRCs, UPF2 was not differentially expressed between tumor and matching normal mucosa (*P* = 0.56 and *P* = 0.83, respectively; paired t-test).

By measuring the levels of UPF1 and UPF2 mRNAs by real-time quantitative RT-PCR in another independent series of MSI primary CRCs for which we also obtained the samples corresponding to matching normal mucosa, we observed an approximately 7-fold over-expression of UPF1 in tumors (n = 25) compared to normal mucosa (*P* = 0.0015; paired t-test) (Figure 2). Since the quantification (7-fold) of the over-expression has to be taken with cautiousness due to the small amount of data, we verified that UPF1 mRNA was indeed over-expressed in MSI primary CRCs by performing a qualitative chi-square test, which is robust to extreme values. Using this approach, the number of cases in which UPF1 expression was higher in tumors compared to matching normal mucosa (N = 20/25) was significantly different than expected by chance (*P* = 0.009; chi-square test). These data are illustrated on Figure 2, in which 20 and 5 open circles representing MSI tumor samples over-expressing UPF1 or not, respectively, are represented above a doted line symbolizing the expression of this NMD factor in normal mucosa. Of interest, a trend for over-expression of this factor was also observed in non-MSI (MSS) CRCs (n = 31) in the same conditions (*P* = 0.08; paired t-test) (Figure 2). In contrast, UPF2 was not differentially expressed in either MSI or MSS CRCs compared to matching normal mucosa (*P* = 0.56 and 0.83, respectively; paired t-test) (Figure 2).

### Significant decay of frameshift mutation-derived mRNAs that depend on UPF1 expression in MSI primary CRCs

**Figure 3:**
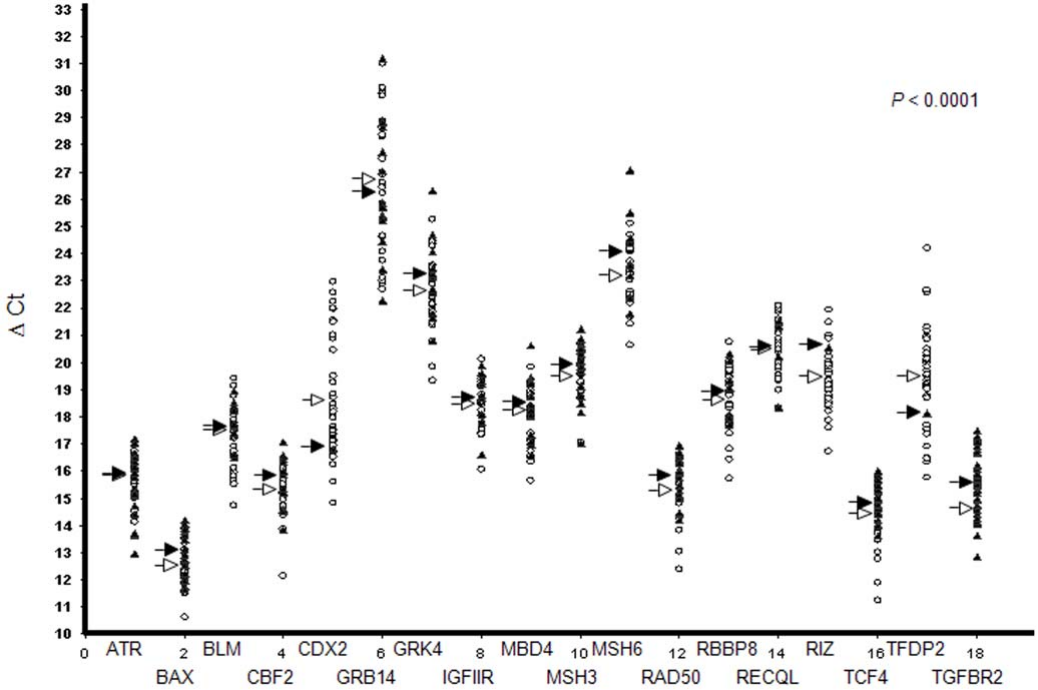
Target gene-related mRNA expression according to mutational status in 44 MSI primary CRC. ∂Ct values are indicated relative to the mutational status of each gene in the 44 MSI primary CRCs (wild-type and mutated tumor samples are indicated by white circles and black triangles, respectively). For each gene, medium values of ∂Ct related to wild-type (white arrow) or mutated (black arrow) tumor samples were calculated. For all genes except 3 (*CDX2*, *GRB14*, *TFDP2*), a trend for decay of mutant compared to wild-type mRNAs was observed (∂Ct values are inversely proportional to gene expression). Overall, the data provide evidence for significant decay of PTC-mRNAs compared to wild-type mRNAs *in vivo* in MSI primary CRCs (*P*<10^−4^, student *t*-test).

In our series of 44 primary MSI CRCs, we further determined the relative *in vivo* expression of 18 MSI target genes by real-time quantitative RT-PCR using experimental conditions that we previously determined in a series of CRC cell lines (*ATR*, *BAX*, *BLM*, *CBF2*, *CDX2*, *GRB14*, *GRK4*, *IGF2R*, *MBD4*, *MSH3*, *MSH6*, *RAD50*, *RBBP8*, *RECQL*, *RIZ*, *TCF4*, *TFDP2*, *TGFBR2*) (Figure 3 and also Table S3 for the mutational status of these 18 genes in MSI CRCs) [14]. For all genes except 3 (*CDX2*, *GRB14*, *TFDP2*), a trend for decay of mutant compared to wild-type mRNAs was observed. The decay of mutant mRNAs compared to wild-types was significant for MSH6 (*P* = 0.02; Student's t-test) and nearly significant for a few others only, e.g. BAX (*P* = 0.08), CDX2 (*P* = 0.10), MSH3 (*P* = 0.10), RIZ (*P* = 0.10) and TFDP2 (*P* = 0.10), providing evidence for differential decay of PTC-mRNAs compared to wild-type in our series of primary CRCs, as already described in a series of MSI CRC cell lines [14] (Figure 3). Overall, there was a highly significant decay of PTC-mRNAs in the MSI primary CRCs (*P*<10^−4^, Student's t-test; see the “Materials and Methods” section for Statistical analyses). As indirect proof of the contribution of NMD, the overall intensity of this process in MSI primary CRCs was positively correlated to the endogenous UPF1 expression level (*P* = 0.02, Student's t-test; see the “Materials and Methods” section for statistical analyses), whereas the impact of UPF2 was not significant (*P* = 0.72, Student's t-test) (data not shown).

### UPF1 and UPF2 are negative predictive factors of the host's immune response against MSI CRCs

With the exception of *TCF4*, no significant positive correlations were found between the endogenous expression of PTC-mRNAs and the presence of CD3ε-positive TILs in tumors (*P*_TCF4_ = 0.06; Bootstrapped t-test) (data not shown). The link between the number of TILs and the expression of UPF1 and UPF2 was not linear but log-linear. Thus, the influence of UPF1 and UPF2 expression on the numbers of TILs was investigated *via* a Wald test and the coefficients in the Poisson regression model were estimated via iteratively re-weighted least squares. Based on these observations, a negative effect of UPF1 and UPF2 expression on the overall number of CD3ε-positive TILs was demonstrated (*P*<0.01 for UPF1 and UPF2; Wald test). A mathematical model correlating the number of CD3ε-positive TILs with the expression of these NMD factors in MSI CRCs was established, predicting that TILs increased by about 30% when the expression of UPF1 was halved.

**Figure 4:**
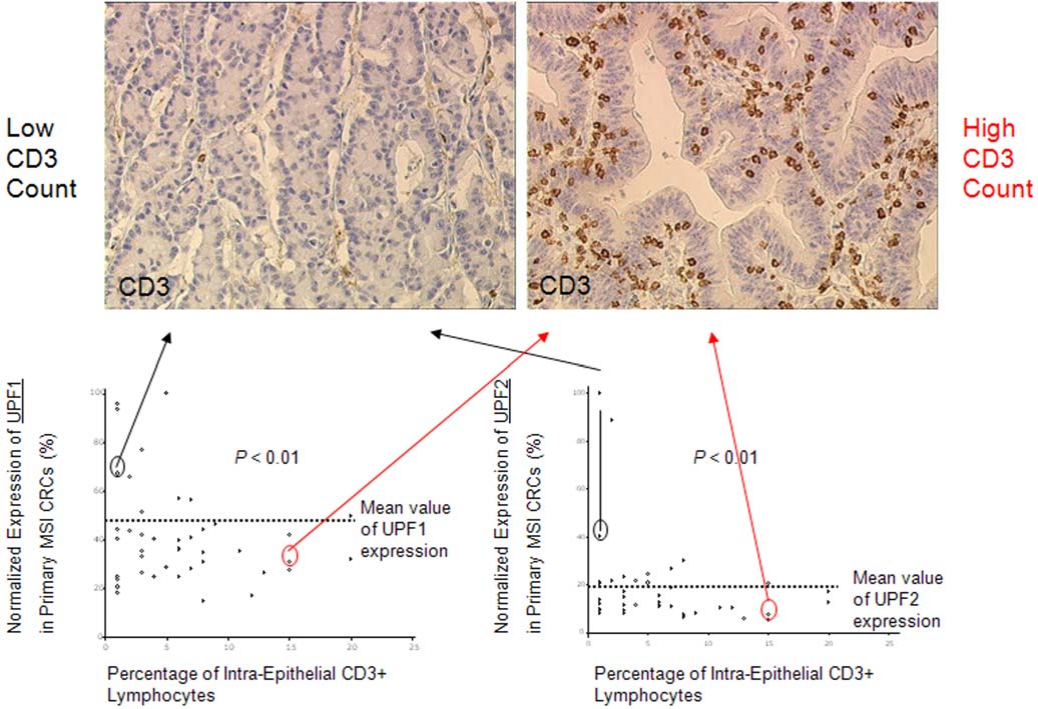
Relationship between endogenous UPF1 and UPF2 mRNA expression in MSI CRCs and the number of TILs. The overall number of CD3ε positive-TILs was significantly related to the endogenous expression of UPF1 and UPF2 in this series of 44 MSI primary CRCs (*P*<0.01; Wald test). Of interest, while tumor samples in which UPF1 and UPF2 expression was low (below the average) presented with variable rates of CD3ε positive-TILs, tumor samples in which UPF1 and UPF2 expression was high (above the average) were almost always characterized with low CD3 count (poorly immunogenic CRCs). These data make UPF1 and UPF2 specific markers of poor anti-tumor immunity in MSI primary CRCs. CD3 protein immunostaining are presented for 2 MSI primary CRC samples showing either low CD3 count and high UPF1 and UPF2 expression (left) or high CD3 count together with low UPF1 and UPF2 expression (right).

These data are illustrated for UPF1 and UPF2 in Figure 4; tumor samples in which UPF1 and UPF2 expression are low (below the average) presented with variable rates of CD3ε positive-TILs while tumor samples in which UPF1 and UPF2 expression are high (above the average) were found to be nearly always characterized by a low CD3 count, making UPF1 and UPF2 possible markers of poor anti-tumor immunity in MSI primary CRCs.

## Discussion

By evaluating a large number of target genes that were mutated at variable rates in their coding repeat sequences, we show a significant decay of the corresponding mutant mRNAs compared to wild-type in MSI primary CRCs with a significant impact of endogenous expression of UPF1 in this process, with UPF2 playing a minor role in the process, as recently described by our group in a series of MSI CRC cell lines [14]. Taken one by one, we observed a significant or nearly significant decay of only some mutant mRNAs compared to wild-types and this is not surprising since: (i) as recently published by our group, NMD impact on the expression of frameshift mutation-derived mRNAs is highly variable from one mutant to another [14]; (ii) frequencies of target gene alterations were highly variable and sometimes very low in our tumor series; (iii) some mutants in particular *(TCF4)* are NMD-irrelevant. In this study, we also investigate large scale gene expression changes in MSI CRC cells in the context of NMD inhibition using a specific method, showing that it led to the up-regulation of 1251 genes in HCT116, among which a proportion of them significantly higher than expected by chance contained a coding microsatellite. Although not all of them are expected to be mutated in MSI CRC cells, as shown here on a short series of up-regulated target genes, it can be advanced that UPF1 is particularly relevant for modulating the expression of dozens of genes mutated in MSI CRC cells. Overall, these data confirm for the first time, both *in vitro* and *in vivo*, that NMD deeply influences MSI-driven tumorigenesis at the molecular level.

As a possible functional consequence of NMD activity *in vivo* in MSI tumorigenesis, we investigated whether the activity of this system modulates anti-tumor immunity. Of interest, the only frameshift mutation whose presence was significantly associated with a higher number of TILs in MSI CRCs was in *TCF4*, the only NMD-irrelevant target gene studied in our series as its coding repeat sequence is located within its last exon. Moreover, an inverse correlation between UPF1 and UPF2 expression and the number of CD3ε-positive TILs was observed in MSI CRCs, and a mathematical model linking the number of CD3ε-positive TILs to the expression of NMD factors in MSI CRCs predicted that TILs increased by about 30% when the expression of UPF1 or UPF2 was halved. These data indicate that NMD may impact negatively the host's immunity against MSI tumor cells in a cumulative manner *via* the contrasted and incomplete degradation of mutated transcripts encoding immunogenic peptides. It could be speculated in this context that UPF factors may be specific markers of poor immunity in MSI primary CRCs. In light of these results, it can thus be advanced that the aforementioned negative impact of NMD on the immune process developed by the host against MSI tumor cells would be efficient only when NMD factors are over-expressed and highly active in degrading the numerous PTC-mRNAs encoding for immunogenic peptides that are synthesized in MSI CRCs. In contrast, it can be assumed that, when expressed at lower rates, NMD factors would be inefficient in preventing the development of an anti-tumor immune response whose intensity would depend on the number and the nature of frameshift alterations accumulated in MSI cancer cells. These findings may be of clinical interest since, as mentioned earlier, anti-tumor immunity is generally considered as an independent factor whose intensity has been consistently demonstrated to be predictive of favorable outcome independently of the initial colon tumor stage and other clinical factors [20].

Since the efficiency of NMD for degrading mutant mRNAs from target genes is highly variable, we recently proposed that NMD may therefore play an important role in the selection of target gene mutations with a functional role in MSI carcinogenesis [14]. It is noteworthy that among the genes up-regulated upon UPF1 silencing, four not yet reported target genes (*SLC35F5*, *TTC3*, *ARV1* and *SMAP1*) are here demonstrated to be frequently mutated in our series of MSI primary CRCs. Amongst them, *SMAP1*, for Stromal Membrane Associated Protein-1, has previously been reported to participate in chromosomal rearrangements with *MLL* in hematological malignancies [21]. With a 73% frequency of frameshift alterations in MSI primary CRCs, including homozygous mutations in five CRC samples (data not shown), it is one of the most frequently mutated target genes in MSI CRCs together with *TGFBR2* and *ACVR2* [17]. In keeping with our previous results and those obtained by Ionov et *al.* using emethine as a nonspecific inhibitor of the NMD system [14], [22], we confirmed in this study that *TGFBR2* PTC-mRNA is degraded through NMD in MSI CRC cells. In a recent paper, You et *al.* [23] reported contradictory data, claiming that this target gene as well as *MSH3* escape NMD in the HCT116 cell line. They presented results on a series of PTC-mRNAs showing these transcripts were either completely sensitive or completely resistant to NMD in MMR-deficient cells. Their data were obtained by performing expression assays that were neither quantitative nor allele-specific. In the same study, these authors also suggested a systematic translational repression of truncated proteins from frameshift mutation-derived mRNAs that escaped NMD (NMTR for “Nonsense Mediated Translational Repression”) [23]. We verified by western blotting that expression of the corresponding proteins for both of the homozygously mutated target genes (*TGFBR2*, *MSH3*) was not detectable in HCT116 cells (data not shown). Contrary to You et *al*, we propose that loss of *TGFBR2* and *MSH3* expression is mainly due to NMD rather than to an hypothetical NMTR pathway. It is noteworthy that the existence of an NMTR pathway does not suit well with the immunogenic properties of MSI cancer cells that depend directly on the accumulation of immunogenic peptides derived from numerous frameshift-related proteins whose PTC-mRNAs are NMD-relevant in most cases [18], [19], [20]. Regardless of discrepancies, all these data argue for a role of NMD in modulating the expression of several genes whose mutations have been already demonstrated *(TGFBR2*, *MSH3*, *MBD4)* or are expected to play an important role during MSI CRC progression [17]. UPF1 depletion with shRNA in non-MSI HeLa cells was recently reported to induce cell cycle arrest in early S phase due to the involvement of this factor in DNA repair [24]. In contrast, transient silencing of UPF1 and/or UPF2 in MSI and MSS colorectal cancer cells (HCT116, LoVo, Co115, LS174T, SW480, COLO320) did not lead to significant alterations of cell growth and/or death in our hands (data not shown). Further studies are now required to determine how NMD activity may change the repertoire of genes involved in MSI carcinogenesis and impact tumor progression using MMR deficient mouse models in which UPF1 activity is modified.

Our observations concern the most frequent primary tumor location associated with MSI in human, i.e. colon. They indicate that NMD deeply influences the expression of numerous mutants with an expected crucial role in MSI-driven tumorigenesis and negatively impacts the host immunity against MSI cancer cells. Such a putative oncogenic function of NMD in MSI carcinogenesis fits well with the fact that we also report here for the first time that UPF1 is significantly over-expressed in MSI CRCs compared to matching normal mucosa. This last observation was based on the measure of the UPF1 mRNA level. As a perspective, it has now to be confirmed at the protein level through a quantitative approach such as Western Blotting that was here not performed because of the lack of available additional tumor material. The development of clinical trials should now be developed to look whether NMD may be considered as a factor of poor prognosis in MSI CRCs or not. Small molecules inhibiting NMD are now available [25] and may be used to gain further insight into the NMD role in cancers. We now plan to use these drugs in MMR-deficient mice developing MSI neoplasms to evaluate whether it may constitute a new therapeutic target for the treatment of such tumors.

## Materials and Methods

### Tumor samples and cell lines

The CRC cell line HCT116 was purchased from the American Type Culture Collection and maintained in DMEM (Life Technologies) containing 10% fetal calf serum (Invitrogen) and Glutamine without antibiotics to allow transient transfection experiments with siRNA. Forty-four MSI primary tumors were obtained from patients undergoing surgery for colorectal cancer; all cases were histopathologically confirmed as being adenocarcinomas. Collected tumors were systematically formalin-fixed, paraffin-embedded and frozen after surgery without any prior embedding in liquid nitrogen. The MSI status was determined by fluorescent multiplex PCR comprising 5 quasimonomorphic mononucleotide repeats (BAT-25, BAT-26, NR-21, NR-24 and NR-27), as described [26]. Only tumors with instability at three or more of these markers were included in the study. The amount of normal contaminating DNA was estimated as previously described [27]. In addition, an independent series of 25 MSI and 31 MSS primary CRC samples were collected in the same conditions together with their matching normal mucosa and used to compare UPF1 and UPF2 expression in tumor and normal colonic tissues.

### Multiplex PCR and mutation analysis

Tumor DNA from fromzen samples was extracted using QIAamp DNA Tissue Kit (Qiagen) according to the manufacturer's instructions. A total of 24 genes containing mononucleotide repeat sequences were chosen either because they were already described as targets for MSI-driven mutations in MSI tumors (e.g. *ATR*, *BAX*, *BLM*, *CBF2*, *CDX2*, *GRB14*, *GRK4*, *IGF2R*, *MBD4*, *MSH3*, *MSH6*, *RAD50*, *RBBP8*, *RECQL*, *RIZ*, *TCF4*, *TFDP2*, and *TGFBR2*) or because they were significantly up-regulated in MSI HCT116 cells following UPF1 silencing and mutated in the HCT116 MSI CRC cell line (*SLC35F5*, *ARV1*, *EFHC1*, *TTC3*, *SMAP1*, *WDR19*). Specific primers for each target gene were designed using e-primer3 (http: // bioweb.pasteur.fr/seqanal/interfaces/eprimer3.html) so that short fragments (<200 bp) could be simultaneously amplified by 6 PCRs using 6-FAM or HEX labeled primers. PCR reactions were performed in a final volume of 20 µl containing 100 ng of genomic DNA, 0.15–0.40 µM of each pair of primers and 1 unit of HotStarTaq DNA polymerase (Qiagen) (Table S4). Adequate dilutions of the fluorescent PCR products were mixed with formamide and GeneScan™ 400HD ROX™ Size Standard (Applied Biosystems), heat-denatured and run on a short capillary containing GS Performance Optimized Polymer 4, on the ABI 3130 Genetic Analyzer using the GeneMapper 3.7 software (Applied Biosystems).

### Real-time Quantitative RT-PCR analysis in primary CRCs

DNA and RNA extractions were performed on closely related regions of each frozen tumor sample. Total RNA was isolated using Trizol reagent according to the manufacturer's instructions (Invitrogen). RNA integrity was evaluated on a 2100 Bioanalyzer using the RNA 6000 Nano LabChip kit (Agilent). Only samples with intact RNAs were used for gene expression analysis (28S/18S RNA ratio >1.6 and absence of aberrant pick on the RNA profile). cDNAs were synthesized using the High Capacity cDNA Archive Kit according to the manufacturer's instructions (Applied Biosystems). For quantitative RT-PCR experiments, expression values of each mRNAs were calculated relatively to 18S ubiquitous RNA as described [14]. Briefly, expression values were obtained from the Ct number at which the increase in signal associated with exponential amplification of PCR products starts to be detected using the Applied SDS Biosystems analysis software according to the manufacturer. Quantification of the 18S ubiquitous RNA was used as the endogenous reference. Results were expressed as N-fold difference in target gene expression relative to 18S expression (∂Ct), where ∂Ct was determined in each case by subtracting the average Ct value of the target gene from the average Ct value of the 18S gene. ∂Ct is inversely correlated to the relative expression values by the formula:
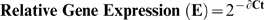



Primers and internal probes for 18S and the target genes were those proposed on demand by Applied Biosystems (TaqMan gene expression assays on demand). For each set of primers, a no-template control and a no-reverse transcriptase control (reverse transcriptase-negative) assays produced negligible signals (usually Ct >35), and were used to confirm the absence of primer-dimer formation and genomic DNA contamination. PCR reactions were performed in triplicate using an ABI Prism 7900 Sequence Detection System and the TaqMan PCR master mix (Applied Biosystems). The thermal cycling conditions comprised an initial denaturation step at 95°C for 10 min and 40 cycles at 95°C for 15 s and 60°C for 1 min.

### Transient transfection assays of cell lines and micro-array analyses

The HCT116 CRC cell line was transiently transfected as described [14]. Microarray experiments were performed by the PartnerChip Company (Evry-France) on Affymetrix GeneChip® Human Exon 1.0 ST arrays. Preparation of single-strand biotinylated cDNA was done according to protocols from the manufacturer (Affymetrix). Briefly, 1 µg of total RNA was subjected to mRNA enrichment using magnetic beads before reverse transcription. Double-stranded cDNA was generated using T7-promoter coupled random hexamers and the Superscript II Reverse Transcriptase. *In vitro* transcription was then carried out in the presence of T7 RNA Polymerase for complementary RNA amplification. At the end, cRNA was reverse-transcribed into single-strand sense cDNA, fragmented and finally biotinylated using terminal deoxynucleotidyl transferase (TdT) before overnight hybridization on Human Exon 1.0 ST arrays. Washes and streptavidin-phycoerythrin (SAPE) staining procedures were performed using Affymetrix Fluidics Station 450 and arrays were finally scanned into Affymetrix Scanner 3000.

### Immunohistochemistry

Tissue from 44 MSI tumors was available. Paraffin-embedded stored tissue was retrieved and fresh 4-µm sections were mounted on silanised slides. Immunohistochemical analysis of CD3 was performed using a CD3 antibody (clone SP7, 1∶300 from Neomarkers, Freemont CA) and the commercially available Bond automated system (Bond™). Quantification of the relative number of CD3+ positive cells was performed in all cases by using a video-assisted measuring system in combination with a software package for quantification (Mercator, exploranova). When assessing TILs, a region of interest was established in an area with cancerous glands which contained the maximal amount of neoplastic cell with minimal stroma or necrotic debris. Automated exploration was done in the region after saving parameters necessary to recognize lymphocytes that reacted positively to the antibodies. The ratio of TILs to epithelial cancerous cells was based on 500 minimal epithelial cells and the result was edited in Microsoft Excel.

### Statistical analysis

#### Normalization of micro-array data and analyses

Quantile normalization was performed using the ExACT software from Affymetrix. Background was calculated and subtracted from main probe intensities using the antigenomic probes, as already described [28]. Only probes with a low DAPG p-value in at least one experimental was selected [28]. Probes that are tagged as “cross-hybridizing” on the Affymetrix design files were eliminated. A paired T-test on the corrected intensities of the selected probes between the UPF1 depletion experiment and the control experiment was performed. Only gene expression alterations above a 1.5 fold-change (up or down) with a p-value ≤0.005 were selected.

#### Testing the effect of the presence of frameshift mutations in target genes on the relative expression of their corresponding mRNAs in MSI primary colorectal tumors

For the 18 target genes *(ATR*, *BAX*, *BLM*, *CBF2*, *CDX2*, *GRB14*, *GRK4*, *IGF2R*, *MBD4*, *MSH3*, *MSH6*, *RAD50*, *RBBP8*, *RECQL*, *RIZ*, *TCF4*, *TFDP2*, *TGFBR2)*, the ∂Ct values measured in the 44 primary tumor samples were standardized. The fitted linear mixed-effects model can be expressed, for each gene *g* and each tumor sample *s*, as: *∂*CtStand*_gs_* = *α*+*β*+*μ_s_*+*ε_gs_* for mutated genes and *∂*CtStand_gs_ = *β*+*µ_s_*+*ε_gs_* for wild-type genes, where ∂CtStand denotes the standardized value of the ∂Ct, *µ_s_* is a random effect depending only on the tumor sample *s* and *ε_gs_* is a random error depending on the tumor sample *s* and the gene *g*. The variance component of the random effect has been estimated by restricted maximum likelihood, *via* the EM algorithm, while the values of *α* and *β* were computed by standard maximum likelihood estimation. In addition, a Student's t-test for the null hypothesis *H*_0_: the presence of the mutation has no effect on the expression of the corresponding mRNA (*α* = 0) versus the alternative *H*_1_:*α*≠0 was performed.

#### Testing the influence of the endogenous expression of UPF1 or UPF2 on the overall decay of mutant mRNAs in MSI primary CRCs

In each tumor sample, the mean of standardized ∂Ct values of mutated genes, denoted by ∂CtStand(mut)_s_, and the mean of standardized ∂Ct values of wild-type genes, denoted by ∂CtStand(wild)_s_, were computed. To test the effect of the preliminary standardized ∂Ct values of UPF1 and UPF2, denoted respectively by ∂CtStand(UPF1)_s_ and ∂CtStand(UPF2)_s_, on the difference between the means for mutated genes and for wild-type genes, the following linear model has been fitted : ∂CtStand(mut)_s_−∂CtStand(wild)_s_ = *α*+*β*·∂CtStand(UPF1)_s_+γ·∂CtStand(UPF2)_s_+*ε_s_*, where again *ε_s_* is a random error depending on the tumor samples. The values of *α* and *β* and *γ* were computed by least-square estimation. Two Student tests were performed: a test for the null hypothesis *H*_0_ : the expression of UPF1 has no effect on the difference of the means (*β* = 0) versus the alternative *H*_1_ : *β*≠0 and a test for the null hypothesis *H*_0_ : the expression of UPF2 has no effect on the difference of the means (*γ* = 0) versus the alternative *H*_1_ : *γ*≠0.

#### Testing the influence of the endogenous expression of UPF1 or UPF2 on the overall number of Tumor Infiltrating Lymphocytes (TILs) in MSI primary CRCs

As the response variable “the number of TILs” is discrete, a classical linear model would not have been appropriated to investigate an effect of UPF1 or UPF2 on it. The most common model in this particular case is the Poisson regression with a log link function. We checked that the log was the best link function. As a consequence, the link between the number of TILs and the expression of UPF1 and UPF2 is not linear but log-linear and the Pearson correlation coefficient is not appropriate. The influence of UPF1 and UPF2 expression on the numbers of TILs was thus investigated *via* a Wald test. Furthermore, the coefficients in the Poisson regression model were estimated via iteratively re-weighted least squares.

## Supporting Information

Table S1 Genes expression data concerning HCT116 cells upon inhibition of UPF1 expression by siRNA. Only genes for which a significant de-regulation was observed (1.5 fold-change up or down with a p-value ≤0.005) in these conditions are listed.
(0.25 MB XLS)

Table S2 List of the target genes containing coding microsatellite sequences and up-regulated following UPF1 silencing in HCT116. In each case, the number of the exon containing the longer coding repeat tract is indicated.
(0.09 MB XLS)

Table S3 Frameshift mutations in 18 target genes whose mRNAs were quantified in 44 MSI primary CRCs. WT: Wild Type; M: Mutated.
(0.04 MB XLS)

Table S4 List of the primers used for the screening of frameshift mutations at coding microsatellite sequences contained in 24 target genes for MSI in CRCs.
(0.03 MB PDF)
