# Supplementary material for: Nonsense-Mediated mRNA Decay Impacts MSI-Driven Carcinogenesis and Anti-Tumor Immunity in Colorectal Cancers
Source: PLoS One. 2008 Jul 9;3(7):e2583. doi: 10.1371/journal.pone.0002583 (PMC2440797; doi:10.1371/journal.pone.0002583)
Supplement: Table S4 — List of the primers used for the screening of frameshift mutations at coding microsatellite sequences contained in 24 target genes for MSI in CRCs. (0.03 MB PDF) [file pone.0002583.s004.pdf]

Primers used for the screening of frameshift mutations at coding microsatellite sequences

Gene	Forward	Reverse	Type of Labelling (reverse primer)
**ATR**	tgaaagcaagttttactggactagg	tcttctgtaggaacttgaaagcc	Hex
**BAX**	agttcgtccccgatgcgcttgaga	ttcatccaggatcgagcagggcga	Fam
**BLM**	acagcagtgcttgtgagaac	ctctgccaccaggaagaatc	Fam
**CBF2**	tcccttactttgtcatcacc	ccatgaagaaagtgaattgg	Hex
**CDX2**	gccaggtctgtaggtctatg	ctcagtgtctggctctgtc	Fam
**GRB14**	gcctgtaccttaaagcagaa	ggcaatagtgatatttatgt	Hex
**GRK4**	tttagagccatagcttcacc	tgctgtttaaactaggtttgc	Fam
**IGF2R**	gagcgctgataaaccttatg	aggctaagtttgacagccta	Fam
**MBD4**	cagaacaaaaatttgatcctgaactc	gatgctggagcatgtggt	Fam
**MSH3**	actcccacaatgccaataaaaa	agatgtgaatcccctaatcaagc	Hex
**MSH6**	cgtaatgcaaggatggcgtg	gggtgatggtcctatgtgtc	Hex
**RAD50**	caagtcccagcatttcatca	aactgcgacttgctccagat	Hex
**RBBP8**	ccaagactgtgatgtgtgaa	gtgtcatctcctgtatttgga	Hex
**RECQL**	gaatcctctaaacactgct	tcaagaacttacggaaagg	Fam
**RIZ**	gtgtttattcaggcttccaa	gcaaaatgtcgtcgaataag	Fam
**TCF4**	gttcaccttgtatgtagcgaa	gcctctattcacagataactc	Hex
**TFDP2**	attcctgagcagaattggta	cagaagaacattaggcgaag	Hex
**TGFBR2**	cacatgaagaaagtctcaccaggc	ctttattctggaagatgctg	Fam
**SMAP1**	ttatcaaaactttgggctgt	cagctgtaagtggttttgc	Hex
**SLC35F5**	gtggggaaacttactgcaactc	tcctaccggtatcaaatagcaga	Fam
**ARV1**	atatattttggatggggaca	atcagcaagagttttccgta	Fam
**EFHC1**	ctccaggagttgcctcctta	gcctcaccagtacagccaaa	Hex
**TTC3**	cagcctagagaaactaagactga	ctcctccattctttcttgtg	Hex
**WDR19**	aattaggttcagctgttgga	attcaagatcagctgggtta	Fam
